# 22q11.2 Deletion Syndrome: Cognitive, Visuomotor, and Adaptive Functioning Followed Longitudinally

**DOI:** 10.1002/brb3.70638

**Published:** 2025-06-17

**Authors:** L. Wallin, C. Gillberg, J. Knutsson, E. Fernell, I. C. Gillberg, E. Billstedt

**Affiliations:** ^1^ Gillberg Neuropsychiatry Centre, Institute of Neuroscience and Physiology, Sahlgrenska Academy University of Gothenburg Gothenburg Sweden; ^2^ Department of Neuropsychiatry Sahlgrenska University Hospital, Region Västra Götaland Gothenburg Sweden; ^3^ Child Neuropsychiatry Clinic Sahlgrenska University Hospital Gothenburg Sweden

## Abstract

**Background:**

Longitudinal studies on cognitive, visuomotor, and adaptive function and their relation to outcomes in adults with the 22q11.2 deletion syndrome (22q11.2DS) are limited.

**Methods:**

This study involved 79 participants (43 females, 36 males) from an original cohort of 100 individuals (58 females, 42 males) with 22q11.2DS, assessed at ages 1–35 years between 1997 and 2006 (T1) and followed up in 2017–2022 (T2), when they were aged 18–50. Clinical, neuropsychological, and adaptive functioning assessments were performed.

**Results:**

At the group level, overall Full‐Scale Intelligence Quotient (FSIQ) remained stable; however, females displayed a significant decline in FSIQ and visuomotor integration (Beery VMI) from T1 to T2. At follow‐up, 19 of 56 (34%) participants had an uneven intelligence quotient (IQ) profile, with most (15/56; 27%) showing a higher Verbal Function Index (VFI) than Perceptual Function Index (PFI). At T1, 10 of 49 participants (20%) had this “uneven IQ profile,” defined as having a higher VFI than PFI (Verbal Comprehension Index [VCI] ≥15 IQ points higher than Perceptual Reasoning Index [PRI]), compared to 14 of 49 (29%) at T2. In the psychosis subgroup (*n* = 8), FSIQ and Verbal Intelligence Quotient (VIQ) showed significant decreases; however, the small sample size limits the validity of these findings. Severe to moderate adaptive function impairments, as measured by the Global Assessment of Functioning (GAF) scale, were observed at T2, with T1 FSIQ predicting GAF at T2.

**Conclusions:**

While group‐level intellectual functioning appeared stable, individual declines were noted. Long‐term follow‐up is essential for personalized support to mitigate severe psychiatric risks, including psychosis with declines in FSIQ, particularly VIQ, potentially indicating or resulting from psychosis in this population.

## Graphical abstract

This longitudinal study examined intellectual, visuomotor, and adaptive function in individuals with 22q11.2 deletion syndrome (22q11.2DS) (*n* = 79) from an original cohort (*n* = 100), assessed at ages 1–35 (1997–2006, T1) and followed up at ages 18–50 (2017–2022, T2). While overall Full‐Scale IQ (FSIQ) in men remained stable, females showed significant declines in FSIQ and visuomotor integration. An uneven IQ profile was more common than in normative groups. In the psychosis subgroup (*n* = 8) at T2, both FSIQ and Verbal IQ (VIQ) had declined from T1. Severe to moderate adaptive impairments (GAF) were observed at T2, with FSIQ at T1 predicting GAF. Long‐term follow‐up is crucial to address psychiatric risks, especially psychosis‐related IQ declines.

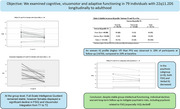

## 1 Introduction

The 22q11.2DS is a genetic disorder caused by a microdeletion on chromosome 22, affecting approximately one in 992 pregnancies (Grati et al. 2015) and one in 2000–4000 live births (Oskarsdottir et al. 2004). It presents with highly variable physical and psychiatric features, including congenital and later‐onset conditions, impacting multiple organ systems. The increased prevalence of neurodevelopmental disorders (NDDs), such as intellectual disability (ID), autism spectrum disorder (ASD), and attention‐deficit/hyperactivity disorder (ADHD), and psychiatric disorders in 22q11.2DS is well established (McDonald‐McGinn et al. 2015). The 22q11.2DS is strongly associated with an increased risk of psychotic disorders—about 25%—with a similar age of onset and symptom profile to other psychotic disorders (Bassett et al. 2003; Karayiorgou et al. 2010). Developmental and functional outcomes depend on the severity of physical anomalies, NDDs, psychiatric disorders, and psychosocial factors such as healthcare access and social support (Boot et al. 2023).

In a previous study on this cohort, we present high rates of NDDs as well as psychiatric disorders (Wallin et al. 2023).

Adaptive skills are the everyday life skills needed for independent living and successful participation in society. These skills are typically categorized into three areas: conceptual, social, and practical. Conceptual skills involve understanding and applying information; social skills relate to interpersonal interactions; and practical skills are those needed for daily living.

Adaptive functioning in adulthood differs widely in 22q11.2DS, with most individuals experiencing adaptive deficits (Leader et al. 2023; Vingerhoets et al. 2024). Adaptive functioning and socioeconomic status (SES) are closely related, as SES significantly influences the development and use of adaptive skills. Deficits in adaptive functioning may affect SES, and lower SES is associated with elevated stress and mental health challenges (Hao and Farah 2020), indicating a reciprocal relationship where low SES adversely impacts adaptive functioning and vice versa.

Cognitive characteristics in 22q11.2DS are intelligence quotient (IQ) levels ranging from moderate ID to average, visual perceptual deficits, often referred to as “uneven IQ profile” or nonverbal learning disorder (NVLD), and executive function (EF) dysfunction (Moberg et al. 2018; Niklasson and Gillberg 2010). These findings, reported in cross‐sectional studies of children and adults with 22q11.2DS (Antshel et al. 2008), indicate that visual perceptual difficulties can cause issues in math, visual EF, and fine motor skills (DeSerisy et al. 2024). Even though studies report visuospatial abilities to be particularly impaired in this population, the meta‐analysis by Moberg et al. found no clear nonverbal learning deficit (Moberg et al. 2018). While verbal skills may superficially appear rather unaffected, which may lead to “masked” disability, increasing the risk for stress, substantial evidence shows verbal/language impairments in this population (Boerma et al. 2023; Persson et al. 2006; Rakonjac et al. 2016; Solot et al. 2019; Van Den Heuvel et al. 2018). Moreover, recent research links language abilities as highly important in psychosis development, including in 22q11.2DS (Solot et al. 2020).

Few longitudinal studies have examined the stability of IQ or “uneven IQ profile” in relation to well‐being in individuals with 22q11.2DS (Fiksinski et al. 2022). In the overall population, a meta‐analysis of longitudinal studies has shown high rank‐order stability of cognitive abilities, peaking around age 20 and remaining high throughout adulthood. At age 20, with a 5‐year test–retest interval, stability was *ρ* = 0.77 (observed) and *ρ* = 0.86 (disattenuated). Stability is lower in younger children (<0.70 before age 4) and higher in late adolescence (≥0.70 for all intervals) (Breit et al. 2024). Studies on cognitive stability during childhood in this population have yielded mixed results, with some studies reporting declines of up to 10 IQ points, particularly in verbal skills while nonverbal and spatial abilities appear more stable (Duijff et al. 2012; Tobia et al. 2018; Vorstman et al. 2015). Other studies found no IQ decline (Antshel et al. 2014; Kates et al. 2019). However, studies of large 22q11.2DS cohorts reveal age‐related declines in Full‐Scale Intelligence Quotient (FSIQ), Verbal Intelligence Quotient (VIQ), and Performance Intelligence Quotient (PIQ) (Fiksinski et al. 2022). Further, early identification of individuals deviating from expected cognitive trajectories is crucial, as these individuals may be at increased risk for clinically significant conditions, such as schizophrenia, which is associated with a more pronounced decline in FSIQ and VIQ (Fiksinski et al. 2022; Vorstman et al. 2015). Intellectual stability from childhood to adulthood is even less studied, but some authors have reported a decline in VIQ with age (Gothelf, Penniman, et al. 2007; Green et al. 2009). Furthermore, individuals with 22q11.2DS are at increased risk of early‐onset neurodegenerative disorders, like Parkinson's disease, which may contribute to cognitive decline (Boot et al. 2023; Butcher et al. 2013).

While longitudinal studies on 22q11.2DS exist, ours is unique due to its extended follow‐up period and focus on the stability of uneven IQ profiles—a previously unexplored aspect. EF dysfunction is common in 22q11.2DS (Fiksinski et al. 2019; Maeder et al. 2016), yet the association between EF dysfunction in childhood and adult adaptive functioning is unclear. Albert et al. (2018) showed that EF dysfunction and childhood adaptive functioning significantly predicted adaptive outcomes and positive psychotic symptoms in a longitudinal study, while Maeder et al. (2016) found no relationship between childhood executive performance and adult adaptive functioning. Fiksinski et al. (2019) found a significant association between EF and functional outcome, suggesting that early EF interventions may improve long‐term adaptive functioning in individuals with or at high psychosis risk.

Deficits in both gross (Roizen et al. 2007; Sobin et al. 2005) and fine motor control, particularly poor eye–hand coordination and graphomotor deficits (Roizen et al. 2010; Van Aken et al. 2007), in 22q11.2DS have been reported. Underdeveloped visual–motor integration (VMI), including its subcomponents, visual perception and motor coordination, is a risk factor for academic underachievement (Carames et al. 2022). Impaired visual–motor processing may also affect social cognition in schizophrenia (Lu et al. 2021). However, whether childhood VMI is associated with adult psychiatric or adaptive outcomes in 22q11.2DS is unclear.

Psychotic disorders are significantly more prevalent in 22q11.2DS compared to the general population (Schneider et al. 2014). Cognitive deterioration during childhood is suggested to increase the risk of developing psychosis later in life, with clear signs of neuropsychological deficits and deviations often preceding its onset (Kates et al. 2015; Pontillo et al. 2019; Vorstman et al. 2015). Lower VIQ and poorer reading skills during childhood have been associated with psychotic disorders in adolescence or early adulthood (Antshel et al. 2017; Gothelf et al. 2007; Kates et al. 2019). Also, verbal decline has been linked to the development of psychotic disorder in 22q11.2DS (Vorstman et al. 2015). However, a systematic review (Jhawar et al. 2021) reported mixed results on whether VIQ decline predicts psychosis, with findings pointing in different directions. Yet, psychotic disorders in 22q11.2DS are associated with lower intellectual functioning, a link not observed in anxiety and mood disorders (Schneider, Schaer, et al. 2014).

A first objective of this study of 100 individuals with 22q11.2DS followed from childhood (T1) to adulthood (T2) was to assess longitudinal outcomes from three perspectives: (i) intellectual function, including stability of FSIQ and uneven IQ profile, (ii) visuomotor function, and (iii) adaptive function and socioeconomic variables. A second objective was to examine if factors at T1 (FSIQ, NDD, and EF) were associated with adaptive functioning in adulthood.

## 2 Methods

### 2.1 Procedure

One hundred participants (58 females, 42 males) were included in the original study in 1997–2005 (T1) at ages 1–35 years. They were all invited for a clinical and cognitive follow‐up study 15–17 years later, in 2017–2022 at ages 18–50 years. Seventy‐nine accepted participation in the clinical follow‐up (T2), of whom 56 underwent cognitive evaluation with the Wechsler Adult Intelligence Scale (WAIS), and 44 were assessed with Beery VMI to estimate visuomotor skills. For information on participants and assessment tools at T1 and T2, respectively, please see Table 1. There was no significant difference in T1 FSIQ between T2 participators (*n* = 55) (*M* = 71.3, *SD* = 15.1) and T2 non‐participators (*n* = 41) (*M* = 69.0, *SD* = 16.4; *t*(94) = −0.729, *p* = 0.47). Nor was there any significant difference in age (*M* = 31.1, *SD* = 0.9 vs. *M* = 31.3, *SD* = 1.3; *t*(98) = 0.188, *p* = 0.851) between the two groups. Reasons for nonparticipation in WAIS were that the assessment was performed digitally and therefore participants were unable to do the test (*n* = 9), a parent rather than the proband participated (*n* = 6), or the participant was too exhausted or declined to participate (*n* = 8). VMI was offered to all who were assessed with WAIS. Twelve of those declined participation in VMI due to tiredness after WAIS testing. Assessments at T1 were performed by a neuropsychologist, a child and adolescent psychiatrist, and a child neurologist, and at T2 by a neuropsychologist and a child and adolescent psychiatrist, all of whom have long clinical experience in the field (Niklasson et al. 2009; Wallin et al. 2023).

**TABLE 1:** Description of study group and assessment tools at T1 and T2.

Timepoint	T1	T2
Study period	1997–2006	2017–2022
Age (mean, *SD*)	11 (7.1)	27 (6.4)
Total (m:f)	100 (42:58)	79 (36:43)
Wechsler‐tested	82 (36:46)	56 (21:35)
Griffiths‐tested	14 (6:8)	0
VMI‐tested	56 (30:26)	44 (31:13)
DSM version	DSM‐IV	DSM‐5
Diagnostic tools	ADOS	M.I.N.I
	Connors	P.A.R.I.S
	FTF	PSI
Adaptive functioning		GAF scale

Abbreviations: ADOS = Autism Diagnostic Observation Schedule; Beery VMI = Beery‐Buktenica Developmental Test of Visual–Motor Integration; DSM‐5 = Diagnostic and Statistical Manual of Mental Disorders; FTF = Five to Fifteen parent questionnaire; GAF = Global Assessment of Functioning; Griffiths = Griffiths Mental Development Scales for Children; M.I.N.I. = Mini International Neuropsychiatric Interview; P.A.R.I.S = Paris Autism Research Sib pair study; PSI = Psychosocial Interview; Wechsler = Wechsler Intelligence Scales.

### 2.2 Study Group

All participants (*n* = 56 [35 females, 21 males]) had received a diagnosis of 22q11.2DS via fluorescence in situ hybridization (FISH). Most participants had different kinds of NDDs and/or psychiatric disorders (Wallin et al. 2023). At follow‐up at a mean age of 27 years (*SD* = 6.4), 21 of 56 (38%) had ASD, 26 of 56 (46%) had ADHD, and eight of 56 (14%) had or had had a psychotic syndrome. Almost half had an anxiety or depressive disorder. The psychotic subgroup participating in intellectual testing included eight participants (five females, three males) at a mean age of 28.8 years (*SD* = 7.8). To be classified in the psychosis group, individuals must either meet criteria for a schizophrenia spectrum disorder or other psychotic disorder (brief psychotic disorder, schizophreniform disorder, schizophrenia, or schizoaffective disorder) or meet criteria for a mood disorder with psychotic symptoms (depressive disorder with psychotic symptoms or bipolar disorder with psychotic symptoms during mood episodes).

Nineteen of 56 participants (34%) used psychoactive medications; however, we did not anticipate negative effects on test results. Medication may rather have improved their psychiatric status, potentially facilitating participation. Medical history and status of participants will be presented in a forthcoming paper.

### 2.3 Instruments Used at T1

For six children, the Griffiths Mental Development Scales for Children (*n* = 6) (Åkerman and Thomassen 1991) were used to evaluate developmental functioning. The Griffiths Mental Developmental Scales comprise six subscales: Locomotor; Personal–Social; Hearing and Language; Eye and Hand Coordination; Performance; and Practical Reasoning. An overall general quotient (GQ) is calculated as the average of Developmental Quotients across all subscales centered around 100 with a standard deviation of 16 (Griffiths 1984).

Intellectual functions were assessed in the remaining group of children/adolescents using (i) the Wechsler Preschool and Primary Scale of Intelligence—Revised (WPPSI‐R) (*n* = 9) (Wechsler 1990), (ii) the Wechsler Intelligence Scale for Children (WISC‐III) (*n* = 33) (Wechsler 1992), and (iii) the WAIS (*n* = 8) (Wechsler 1996). Due to the longitudinal study design, different versions of the Wechsler scales were used; therefore, only verbal functioning/VIQ, perceptual functioning/PIQ, and FSIQ are reported from T1.

The Five to Fifteen (FTF) parent questionnaire (Kadesjö et al. 2004) assesses child development and behavior, with good psychometric properties (Kadesjö et al. 2004; Lambek and Trillingsgaard 2015). It covers areas related to executive functioning, including its subdomains of attention, hyperactivity/impulsivity, passivity, and planning/organizing, and was used to screen for EF dysfunction and externalizing/internalizing symptoms at T1. Scores above the 90th percentile of the norm sample indicate EF dysfunction.

### 2.4 Instruments Used at T2

The WAIS‐IV (Weschler Adult Intelligence Scales) (Wechsler 2008b) were used to assess T2 intellectual functioning. For practical reasons, the Verbal Comprehension Index (VCI) was estimated from the two subtests, Vocabulary and Similarities, and the Perceptual Reasoning Index (PRI) from the subtests Block Design and the Matrix. The estimated scores are available in the WAIS‐IV manual. The regular subtests in the Working Memory Index (Digit Span and Arithmetic) and in the Processing Speed Index (Coding and Symbol Search) were administered. “Uneven IQ profile,” indicating NVLD, is characterized by a discrepancy between higher verbal function and lower visuospatial skills. However, an uneven IQ profile can go both ways, that is, PIQ/PRI > VIQ/VCI. We defined uneven IQ as a difference of ≥15 between the VIQ/VCI and PIQ/PRI measures of the Wechsler scales. Seven participants (13%) had a PIQ > VIQ by 10 IQ points or more, and four of these seven had a difference of ≥15 IQ points.

The Beery–Buktenica Developmental Test of VMI (Beery and Beery 2010) is a standardized instrument measuring VMI skills in individuals aged 2–100. It evaluates the coordination of visual perception and motor skills using 24 geometric forms for copying. The Beery VMI was the only test instrument used at both time points (T1 and T2). One study reported moderate test–retest reliability, with correlation coefficients ranging from 0.54 to 0.58 (Harvey et al. 2017).

A semistructured interview form about life situation, developed by our group (Helles et al. 2017), was used to interview participants and/or parents on various life factors and functional outcomes, including housing and employment status, social connections, marital status, driving license acquisition, lifestyle factors, social support, and overall well‐being.

The Global Assessment of Functioning (GAF) scale (APA 2013) was used to assess the severity of psychiatric symptoms and overall functioning across social, occupational, and psychological domains. Total scores range from 0 to 100, with lower scores indicating greater impairments and higher scores reflecting better functioning. The GAF scores, rated by the research group at a joint conference, were based on all available information. Scores between 51 and 60 indicate moderate difficulties, scores between 41 and 50 reflect more serious problems, and scores below 40 indicate severe symptoms and impairments.

Psychiatric and NDD diagnoses were determined according to DSM‐5, based on all available data, including clinical assessment; diagnostic interview using M.I.N.I. (Sheehan et al. 1998); the P.A.R.I.S schedule, developed by our research group to examine development and comorbidities; and information from a parent/proxy.

Questionnaires used were (i) the Adult ADHD Self‐Report Scale (ASRS) (Kessler et al. 2005), (ii) the Fear Survey Schedule‐III (FSS‐III) (Wolpe and Lang 1964), and (iii) the parent questionnaire Adult Behaviour Checklist (ABCL) (Achenbach et al. 2017). Furthermore, assessment of emotional processing using an eye‐tracking device (Galazka et al. 2023) and collection of blood samples for genetic and amino acid studies were conducted (Wallin et al. 2023).

### 2.5 Statistical Analyses

Chi‐square tests were used for categorical variables, and independent samples *t*‐tests were used to compare sex differences at T2. For continuous variables, nonparametric Mann–Whitney *U* tests (between‐group comparisons) and Wilcoxon sign rank tests (within‐group comparisons) were used. Effect sizes were calculated using Eta squared. All analyses were two‐tailed with a significance level set of *p* < 0.05. To adjust for multiple comparisons and control the family‐wise error rate, the Bonferroni correction was applied, resulting in an alpha value of 0.005. A chi‐square goodness of fit test compared the proportion of “uneven IQ” profiles in the 22q11.2DS group with that in the norm group. Pearson's correlation coefficient assessed the relationship between FSIQ at T1 and T2. Linear regression analyzed whether FSIQ at T1 predicted GAF at T2, controlling for NDD and EF at T1 and for gender and age at T2. All analyses were conducted in SPSS version 29.0.

### 2.6 Ethical Considerations

All study procedures adhered to ethical standards set by national and institutional committees. The T1 study was approved by the Research Ethics Committee at the Faculty of Medicine, Gothenburg (reference: L604‐97). The T2 study was approved by the Regional Ethical Approval Board in Gothenburg (reference: 487‐16).

### 2.7 Participant Consent Statement

Informed consent was obtained from parents or participants at T1 and from all participants at T2, except for three with very low adaptive and cognitive function, for whom consent was provided by a caretaker.

## 3 Results

### 3.1 FSIQ at T1 and T2

**FIGURE 1:**
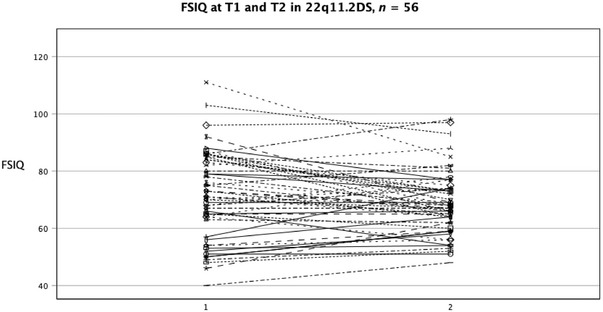
FSIQ at T1 and T2 in 56 individuals with 22q11.2DS. FSIQ = Full Scale Intelligence Quotient.

The mean FSIQ at T2 was 68.2 (*SD* = 11.6, range 40–98) for the 56 participants who took the WAIS test at T2, with no significant sex difference (Table 2). Regarding the group tested on both occasions (*n* = 55), FSIQ was stable for the total group (Figure 1) and for males, while females showed a significant decrease in median (*Md)* FSIQ over time (*Md* = 75.0 vs. 68.00, *z* = −3.039, *p* = 0.002), with a medium effect size (*r* = 0.36). There was also a decrease in VIQ/Verbal Function Index (VFI) for females (*Md* T1 = 84.0, *Md* T2 = 75.0, *z* = −2.553, *p* = 0.011), with a medium effect size (*r* = 0.33); however, this decrease was not significant when adjusting for multiple analysis. No significant change was found in PIQ/Perceptual Function Index (PFI). There was a strong correlation between FSIQ at T1 and FSIQ at T2 (*r* ^2^ = 0.58 [CI = 0.613–0.850], *n* = 55, p < 0.001).

**TABLE 2:** Adaptive, cognitive, and visual–motor integration function at T2.

	Total (*n* = 56) Mean (*SD*)	Range	Male (*n* = 21) Mean (*SD*)	Female (*n* = 35) Mean (*SD*)
Age	27.0 (6.4)	18–50	28.1 (5.7)	26.4 (6.8)
GAF	50.5 (11.6)	25–72	52.1 (9.3)	49.5 (12.8)
FSIQ	68.2 (11.6)	40–98	66.8 (8.0)	69.0 (13.3)
VCI	74.2 (12.3)	50–106	74.1 (10.1)	74.3 (13.6)
PRI	69.6 (11.7)	50–100	67.9 (9.2)	70.6 (13.1)
WMI	72.3 (11.0)	50‐99	71.2 (8.6)	73.0 (12.2)
PSI	75.6 (13.0)	50‐112	73.2 (10.6)	77.0 (14.2)
Beery‐VMI, *n* = 44	53.4 (10.2)	45‐101	49.5 (6.2)	59.3 (16.7)
Beery‐VMI*p*, *n* = 42	70.1 (13.8)	45‐94	69.3 (11.5)	70.5 (14.7)
Beery‐VMI*m*, *n* = 41	68.0 (18.8)	45‐97	65.5 (16.3)	69.0 (19.8)

Abbreviations: Beery‐VMIm = Beery Visual–Motor Integration motor and coordination; Beery‐VMIp = Beery Visual–Motor Integration perception; Beery‐WMI = Beery Visual–Motor Integration; FSIQ = Full Scale Intelligence Quoting; GAF = Global Assessment of Functioning; PRI = Perceptual Reasoning Index; PSI = Processing Speed Index; VCI = Verbal Intelligence Quoting; VMI = Working Memory Index.

Overall, 17 individuals demonstrated a change of ≥10 IQ points between assessments. This included five individuals who showed an increase in IQ scores and 12 who experienced a decrease. Among those with a large shift, exceeding ≥15 IQ points, two individuals showed improvement, one from 57 to 74 and another from 46 to 62. The remaining six individuals with significant IQ changes exhibited a decrease. Notably, all individuals who experienced a decline in IQ scores were females, while the two who showed improvement were males.

### 3.2 “Uneven IQ Profile” at T1 and T2

Of the 56 individuals tested, 50 were evaluated with Wechsler scale tests at T1 and WAIS at T2, allowing for the study of the stability of “uneven IQ profile”/NVLD. At T1, 10 participants (20%) had had an “uneven IQ profile,” defined as having a higher VFI than PFI (VCI ≥15 IQ points higher than PRI), with five retaining this profile at follow‐up (Table 3). AT T2, nine of 39 participants who had an “even IQ profile” at T1 showed an “uneven IQ profile.”

**TABLE 3:** Stability in uneven IQ profile between T1 and T2, *n* (%).

	Uneven IQ profile T2		
	Even IQ *n* = 35 (71)	Uneven IQ *n* = 14 (29)	Total *n* = 49 (%)
**Uneven IQ profile T1**			
Even IQ, ***n*** = **39 (80)**	**30 (61)**	9 (18)	39 (80)
Uneven IQ, ***n*** = **10 (20)**	5 (10)	**5 (10)**	10 (20)
Total, *n* = 49 (100)	35 (71)	14 (29)	49 (100)

*Note*: Uneven IQ profile is defined as a higher VFI than POI (VCI ≥15 IQ points higher than PRI). The bold formatting is intending to make a clear distinction between T1 and T2 variables.

Abbreviation: IQ = intelligence quotient.

At T2, 19 of 56 participants (34%) had an uneven WAIS profile, primarily with a higher VFI (*n* = 15, 27%). Four participants had a PIQ > VIQ by 15 IQ points or more. The prevalence of this VCI > PRI profile in the IQ corresponding norm group (FSIQ <79) is 13.5% per the WAIS technical manual (Wechsler 2008b). A chi‐square goodness of fit test indicated a significant difference in the proportion of “uneven IQ profiles” in the 22q11.2DS group (27%) compared to this norm group (13.5%, *χ* ^2^(1, *n* = 55) = 8.934, *p* = 0.003).

### 3.3 VMI at T1 and T2

VMI significantly decreased from T1 (*Md* = 61.5) to T2 (*Md* = 47.0, *z* = −2.943, *p* = 0.001). In females, the median VMI score decreased from T1 (*Md* = 75) to T2 (*Md* = 58, *z* = −3.141, *n* = 19, *p* = 0.002) with a large effect size (*r* = 0.51), while no significant change was observed in males. Mann–Whitney *U*‐test showed no significant difference in T2 VMI between males (*Md* = 47, *n* = 13) and females (*Md* = 58, *n* = 31, *U* = 263, *z* = −1.67, *p* = 0.103). At follow‐up, 12 participants (40%) had a decrease of ≥15. Only one participant from the psychotic subgroup had VMI data for both timepoints, limiting stability analysis in that group.

The Bonferroni correction was applied to adjust for multiple comparisons (10 analyses), resulting in an adjusted alpha level of 0.005 (0.05/10). In our study, significance was seen regarding (a) the decline in FSIQ and VMI from T1 to T2 in females, (b) the significant correlation between FSIQ at T1 and T2, and (c) the frequency of “uneven IQ profile” in individuals with 22q11.2 DS (at T2), which is significant compared to an IQ‐matched normative group. On the other hand, there were no significant differences in WAIS or VMI between the psychosis and non‐psychosis groups.

### 3.4 Subsample With Psychosis

**FIGURE 2:**
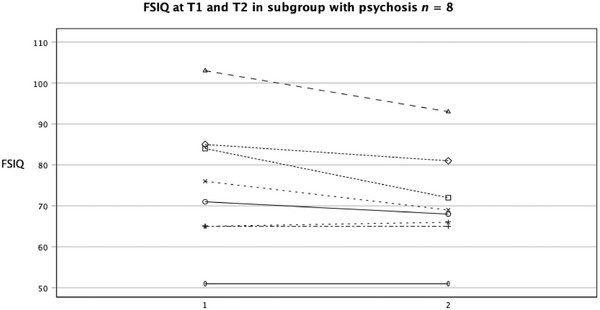
FSIQ at T1 and T2 in the subgroup with psychosis. FSIQ = Full Scale Intelligence Quotient.

FSIQ significantly decreased in the psychosis subgroup (*n* = 8) from T1 (*Md* = 73.5) to T2 (*Md* = 68.5, *z* = −1.992, *p* = 0.046), with a small to medium effect size (*r* = 0.498) and a mean decrease of 4.38 (95% CI: 0.31–8.45) (Figure 2). VIQ significantly decreased from T1 (*Md* = 86.0) to T2 (*Md* = 79.0, *z* = −2.380, *p* = 0.017), with a large effect size (*r* = 0.595) and a mean decrease of 8.75 (95% CI: 2.47–15.03). There was no change in PIQ from T1 (*Md* = 68.5) to T2 (*Md* = 68.0, *z* = −0.281, *p* = 0.779). No statistical analyses were performed on the associations between T1 factors and T2 outcomes due to the small sample size; however, a descriptive overview is presented in Table 4.

**TABLE 4:** T1 and T2 factors in the group with psychotic symptoms.

No/sex	FSIQ T1	FSIQ T2	VIQ‐PIQ ≥15 T1	VIQ‐PIQ ≥15 T2	VMI T2	VMI*p*	VMI*m*	Int	Ext	EF	GAF
1/F	71	68	—	—	45	65	77	NA	NA	NA	63
2/F	84	72	—	—	101	94	92	—	—	—	61
3/M	76	69	—	+	52	61	81	—	—	—	57
4/F	103	93	—	—	101	94	95	—	—	—	70
5/M	65	65	—	—	NA	NA	NA	+	+	+	27
6/F	51	51	+	—	NA	NA	NA	+	+	+	26
7/F	85	81	+	+	45	61	73	NA	NA	NA	33
8/M	65	66	+	—	NA	NA	NA	+	+	+	42

Abbreviations: EF = Executive Function deficits above the 90th percentile; Ext = Externalizing symptoms above the 90th percentile; FSIQ = Full Scale Intelligence Quote; GAF = Global Assessment of Function; Int = Internalizing symptoms above the 90th percentile; NVLD = Nonverbal Learning Disorder; PIQ = Perceptual Intelligence Quote; VIQ = Verbal Intelligence Quote; VMI = Beery Visual–Motor Integration; VMI*m* = Beery Visual–Motor Integration motor and coordination; VMI*p* = Beery Visual–Motor Integration perception.

### 3.5 Adaptive Function and Socioeconomic Factors at T2

Most of the study group required considerable support in daily life, including education, employment, and economy. The mean GAF score at T2 was 50.5 (*SD* = 11.6, range 25–72), indicating moderate to severe psychiatric symptoms and impairment in social, educational/occupational, and psychological functioning (Table 5).

**TABLE 5:** Self‐reported sociodemographic characteristics.

	Total, *n* = 79	Male, *n* = 34	Female, *n* = 45
Age, mean (*SD*)	27 (6.6)	28 (6.3)	27 (7.0)
Age moved from home, mean (*SD*), *n* = 44	22 (4.1)	22 (4.2)	22 (4.0)
Living status (%), *n* = 79			
Living in own household with no support	16 (20)	7 (19)	9 (21)
Living in own household with support	14 (18)	10 (28)	4 (9)
Living with parent	34 (43)	14 (39)	20 (47)
Residential/sheltered living	15 (19)	5 (14)	10 (23)
Marital status^a^ (%)			
Single	61 (77)	30 (83)	31 (72)
Partner	18 (23)	6 (17)	12 (28)
Has close friends^b^ (%), *n* = 72	52 (67)	23 (66)	29 (67)
Occupation status (%), *n* = 79			
Sheltered/supported employment	54 (68)	26(72)	28 (65)
Competitive employment	1 (1)	1 (3)	0
Studies	11 (14)	3 (8)	8 (19)
No occupation	13 (16)	6 (17)	7 (16)
Educational attainment status (%), *n* = 78			
Upper secondary school	37 (47)	14 (39)	23 (55)
Primary school/compulsory school	1 (1)	1 (3)	0
Special school/special upper secondary school	40 (51)	21 (58)	19 (45)
Extra support in school (%), *n* = 77	71 (92)	32 (94)	39 (91)
Financial management (%), *n* = 79			
Manages own finances	19 (24)	7 (19)	12 (28)
Manages own finances with support^c^	41 (52)	19 (53)	22 (51)
Trustee	19 (24)	10 (28)	9 (21)

^a^ Self‐reported “have you ever been in a long‐term romantic relationship (more than 3 months).”

^b^ Self‐reported “persons whom you spend time with on a regular basis and feel that you can trust.”

^c^ Support from parents.

### 3.6 Predictors on Adaptive Functioning

Multiple linear regression was used to assess the impact of T1 FSIQ, NDD, EF, age at T2, and gender on GAF at T2. Results indicated a significant association between T1 FSIQ and T2 GAF. However, the adjusted *r* ^2^ showed that the overall model explained only 8.2% of the variability in GAF. The Bonferroni correction was applied to adjust for multiple comparisons (five analyses), resulting in an adjusted alpha level of 0.01 (0.05/5) (Table 6).

**TABLE 6:** Multiple linear regression predicting Global Assessment of Functioning (GAF): Unstandardized *B* coefficients (*SE*) (dependent variable: GAF).

	Model 1	Model 2	Model 3	Model 4	Model 5
FSIQ T1	0.262^**^ (0.091)	0.250^**^ (0.093)	0.246^**^ (0.096)	0.268^*^ (0.114)	0.310^**^ (0.118)
NDD T1		−1.833 (2.980)	−1.628 (3.146)	−1.684 (3.175)	−2.444 (3.213)
EF T1			−0.696 (3.156)	−0.753 (3.186)	0.141 (3.244)
Age T2				0.111 (0.309)	0.106 (0.307)
Gender					−3.969 (3.114)
Intercept	32.021^***^ (6.598)	33.505^***^ (7.060)	34.150^***^ (7.697)	29.583 (14.932)	28.533 (14.869)
*N*	59	59	59	59	59
*R* ^2^ (adj.)	0.112	0.102	0.086	0.072	0.082

Abbreviations: EF = Executive Function; FSIQ = Full Scale Intelligence Quotient; NDD = Neurodevelopmental Disorder.

*p < 0.05; **p < 0.01; ***p < 0.001.

## 4 Discussion

This prospective study on cognitive, visuomotor, and adaptive functioning and their relations to psychiatric issues in adults with 22q11.2DS followed from childhood highlights the need for ongoing adjustment of support and requirements.

In our group, intellectual functioning ranged from moderate ID to average FSIQ, with most scoring at borderline or mild ID levels, consistent with other studies on adults with the deletion (Bassett et al. 2005; Butcher et al. 2012). While some participants showed an “increase” in FSIQ, most experienced a “decrease,” though differences were statistically nonsignificant. This contrasts with some longitudinal studies reporting significant IQ declines, especially in VIQ, with age (Gothelf, Penniman, et al. 2007; Green et al. 2009) but aligns with one other study (Kates et al. 2019). There was no male:female difference at follow‐up, despite females performing significantly better than males at T1 (Niklasson and Gillberg 2010), consistent with the results from another study (Antshel et al. 2005). These findings suggest that males’ cognitive ability is more affected in childhood in 22q11.2DS, whereas females, perhaps, do show a cognitive decline with age. In our study, females, but not males, showed significant declines in both FSIQ and VIQ.

Visuomotor skills, often deficient in individuals with “uneven IQ profile” (VIQ > PIQ), were relatively poor at T1 and declined further by T2 to a mean VMI of 56, very low compared to the reference mean of 90–109 (Beery and Beery 2010). While we have not found studies specifically on visuomotor performance in adults with 22q11.2DS, the possibility that visuomotor impairments might impact daily living skills highlights the need for awareness of visuomotor problems in this population.

An “uneven IQ profile” was prevalent, but only half of those with this profile at T1 maintained it at T2 (Niklasson and Gillberg 2010), in line with recently updated clinical guidelines on managing adults with 22q11.2DS (Boot et al. 2023). Our sample had a significantly higher prevalence (27%) of individuals with a VCI exceeding their PRI (VCI ≥15 IQ points higher than PRI) compared to the normative group. This finding is noteworthy, as the prevalence of NVLD in American children and adolescents without ASD is estimated at 3%–4% (Margolis et al. 2020). This observed cognitive profile, with stronger verbal skills, may lead to overestimating abilities and overlooking challenges, highlighting the need for further investigation into its implications for our population.

In the psychosis subgroup (*n* = 8), FSIQ scores significantly declined over time, with a greater decline in VFI compared to FSIQ, consistent with prior research (Gothelf et al. 2013; Schneider, Schaer, et al. 2014; Vorstman et al. 2015). Research indicates a strong link between language abilities and the development of psychosis, also in 22q11.2DS (Solot et al. 2020). Studies show age‐related decline in language abilities, which is more pronounced in individuals who have experienced psychotic symptoms, while FSIQ changes may be partially due to VFI alterations; PIQ remained stable over time. The disorganized speech and the poverty of speech observed in psychosis might well correlate with the verbal decline measured with IQ tests. However, some studies on 22q11.2DS report that low baseline IQ predicts the development of psychosis (Schneider, Schaer, et al. 2014), which was not the case for the majority of our group. The small number of participants meeting the study criteria for psychosis (*n* = 8) raises concerns about the validity of our findings regarding intellectual functioning and psychosis development. Further research with larger samples is needed to explore this relationship. Two interpretations from a diathesis–stress perspective arise: (*i)* Higher verbal ability might increase vulnerability to stress due to heightened expectations and insufficient support, or (ii) it may enhance reflective capacity, increasing awareness of stress and vulnerability to psychosis. By T2, the difference in VFI/VCI between groups disappeared, whereas PIQ/PRI and FSIQ did not differ significantly between the psychosis and the non‐psychosis group, neither at T1 nor at T2. Taken together, our results suggest that declines in FSIQ and VIQ may indicate vulnerability to psychosis or be a consequence of it.

While most remained single, 24% reported having a partner, consistent with findings from other studies (Mosheva et al. 2018). Many reported having close friendships, though plausible that some participants included other important people, such as siblings or caregivers in their definitions of friendship, this indicates strong social connections despite the challenges associated with 22q11.2DS.

The wide age range of participants (18‐50 years) is a potential confounding factor. Regarding intellectual functioning, while not anticipated to significantly affect results, the relatively small sample size limits our ability to detect differences in intellectual functioning between age groups. Further, younger participants, around age 18, are at increased risk of developing psychosis due to ongoing neurodevelopmental processes at this age (Schultze‐Lutter et al. 2020). Although the mean age of the psychosis group does not differ numerically from the overall mean age (both 27 years), the broad age range introduces potential confounding effects related to the prevalence of psychosis and varying psychosocial situations. Further analysis, including stratification by age group and perhaps controlling for psychosocial variables, is recommended to assess the influence of age on the observed results.

We found a significant association between T1 FSIQ and variability in functioning at follow‐up. This is in line with Butcher et al. (2012) but contrasts with other studies (Vangkilde et al. 2016; Wagner et al. 2017) that found no significant link between childhood IQ and social functioning in young adulthood. Notably, our assessment of functioning mainly used the GAF scale. Future investigations should employ systematic assessments with adaptive functioning scales for a more comprehensive understanding.

### 4.1 Strengths and Limitations

Even though this is one of the largest among the few longitudinal studies on cognitive, adaptive, and functional development from childhood to adulthood in 22q11.2DS, there are some limitations that need to be taken into consideration. The longitudinal study design is effective in determining variable patterns of symptoms and functioning over time to understand lifespan trajectories. The challenge is that this research design needs to be conducted over a period of decades rather than years. The current study covers about two decades. The drawback of longitudinal studies, however, is the matter of considerable attrition. The resulting relatively small sample size renders difficulties in determining significant differences, especially between subsample measures, as well as in identifying specific predictors for functioning. The lack of an IQ‐matched comparison group is a limitation, but the longitudinal design made it difficult to find an appropriate group. On the other hand, the use of tests with well‐validated norms allowed for some reasonably valid assumptions. Another limitation is that IQ tests are not stable at very young ages; the majority of the study group were above the age of 6 years at T1, whereas six individuals were below the age of 6 years. The change from WISC to WAIS between timepoints might show a change in IQ scores due to different normative comparison groups across the two tests. Present data indicate that WAIS tend to result in somewhat higher scores, so the change from WISC to WAIS could be assumed to have “minimized” the global IQ decline seen on the whole, rather than overstate it (Kaufman and Lichtenberger 2006). Even though there was attrition, the group followed up was considered representative given that there was no difference with regard to FSIQ at T1, gender, or age at T2 between the participating and the non‐participating groups.

### 4.2 Conclusions and Clinical Implications

Our findings underscore the importance of ongoing monitoring of functioning in individuals with 22q11.2DS. Given the prevalence of intellectual and functional impairments, regular clinical assessments are recommended to comprehensively assess cognitive abilities and adaptive and executive functioning and to screen for psychiatric disorders and NDDs. This approach allows for tailored interventions and support, which may require adjustments over time, to promote optimal health and development in this population.

## Author Contributions

C. Gillberg, E. Fernell, C. Gillberg, E. Billstedt, and L. Wallin participated in the planning, conceptualization, and design of the study. E. Billstedt, J. Knutsson and L. Wallin were responsible for acquiring the clinical data and conducting the statistical analyses. L. Wallin drafted the initial manuscript. All authors contributed to and approved the final version of the manuscript.

## Ethics Statement

The original study was approved by the Research Ethics Committee at the Faculty of Medicine, Gothenburg (reference: L604‐97), and the follow‐up study was approved by the Regional Ethical Approval Board in Gothenburg (reference: 487‐16).

## Consent

Informed consent was obtained from parents or participants at T1 and from all participants at T2, except for three with very low adaptive and cognitive function, for whom consent was provided by a caretaker.

## Conflicts of Interest

The authors declare no conflicts of interest.

## Peer Review

The peer review history for this article is available at https://publons.com/publon/10.1002/brb3.70638

## Data Availability

The data that support the findings of this study are available on request from the corresponding author. The data are not publicly available due to privacy or ethical restrictions.
